# Prevalence of Vancomycin-resistant enterococci (VRE) in Egypt (2010–2022): a systematic review and meta-analysis

**DOI:** 10.1186/s42506-023-00133-9

**Published:** 2023-04-11

**Authors:** Ahmed Azzam, Hoda Elkafas, Heba Khaled, Ahmed Ashraf, Mohammed Yousef, Aya Awny Elkashef

**Affiliations:** 1 Department of Microbiology and Immunology, Faculty of Pharmacy, Helwan University, Ain Helwan, Cairo, Egypt; 2 Department of Pharmacology and Toxicology, Egyptian Drug Authority, Formerly National Organization for Drug Control and Research, Cairo, 35521 Egypt; 3 Department of Biochemistry, Faculty of Pharmacy, Cairo University, Cairo, Egypt; 4 Faculty of Pharmacy, Minia University, Minia, Egypt; 5 Faculty of Pharmacy, Al-Azhar University, Assiut, Egypt; 6 Department of Botany & Microbiology, Faculty of Science, Arish University, North Sinai, Egypt

**Keywords:** Vancomycin-resistant Enterococci, Linezolid, VanA, VanB, Egypt, Systematic review

## Abstract

**Background:**

Vancomycin-resistant Enterococci (VRE) represent a critical medical and public health concerns due to their association with serious nosocomial infections and a high risk of mortality. We aimed to reveal the pooled prevalence of VRE and antimicrobial resistance profiles among enterococci clinical isolates in Egypt.

**Methods:**

A PubMed, Scopus, Google Scholar, and Web of Science literature search was carried out in accordance with the Preferred Reporting Items for Systematic Reviews and Meta-Analyses (PRISMA) guideline. Only published studies documenting the prevalence of VRE between 2010 and 2022 were included. Using the random effects model and the 95% confidence intervals, the pooled estimate of VRE was calculated by MedCalc Version 20.113. Cochran’s *Q* and *I*^2^ tests were used to evaluate the degree of heterogeneity, and publication bias was examined by visually examining the funnel plot and its associated tests (Begg’s and Egger’s tests).

**Results:**

The pooled prevalence of VRE among enterococci clinical isolates in Egypt was estimated to be 26% (95% CI 16.9 to 36.3). *E. faecalis* had a greater pooled prevalence than *E. faecium*, with 61.22% (95% CI 53.65 to 68.53) and 32.47% (95% CI 27 to 38.2), respectively. The VanA gene is more frequent than the VanB gene among VRE, with a pooled prevalence of 63.3% (95% CI 52.1 to 73.7) and 17.95% (95% CI 7.8 to 31), respectively. The pooled resistance rate of linezolid was substantially lower than that of ampicillin and high-level gentamicin (HLG) 5.54% (95% CI 2.33 to 10%), 65.7% (95% CI 50.8 to 79.2%), and 61.1% (95% CI 47.4 to 73.9), respectively.

**Conclusion:**

The prevalence of VRE is alarmingly high in Egypt. It is imperative that antimicrobial stewardship activities and infection control programs are strictly adhered to and implemented to prevent further escalation of the problem.

**Supplementary Information:**

The online version contains supplementary material available at 10.1186/s42506-023-00133-9.

## Background

The rise of antimicrobial resistance and the resulting scarcity of effective antibiotics are two global concerns [1]. Nosocomial infections pose a serious risk to people everywhere, and the improper treatment with broad-spectrum antibiotics encourages the spread of hospital-associated multi-drug resistant pathogens [2]. Enterococci are important nosocomial pathogens. They are considered the primary cause of nosocomial infections in the USA and the second-highest cause of such infections globally [3]. They are facultative anaerobic Gram-positive cocci organisms belonging to the lactic acid bacteria. In 1984, group D streptococci were separated from the streptococci and were recognized as a distinct genus, which was named Enterococcus [4]. As per the LPSN List of Prokaryotic Names with Standing in Nomenclature, there are currently 80 verified species within the Enterococcus genus [5]. There are numerous diverse environments where enterococci may be found, including soil, water, on plants, and in the gastrointestinal tracts of both humans and animals [6]. It is a also frequently found in animal-derived foods such as meat, fermented and cooked meats, and cheese [7, 8].

Vancomycin is one of the therapeutic options for enterococci infections, acting by inhibiting peptidoglycan cross-linking by binding to the terminal D-Ala-D-Ala pentapeptide that compromises the integrity of the peptidoglycan layers, eventually leading to cell death [9]. As a tool to facilitate antibiotic stewardship and optimal use, the WHO Model List of Essential Medicines classified antibiotics into Access, Watch, and Reserve (AWaRe) categories for the treatment of priority bacterial infections. Based on the WHO AWaRe classification, vancomycin is classified within the Watch group, which includes most of the “highest-priority critically important antimicrobials” for human medicine and veterinary use. Antibiotics within the watch category are recommended only for specific limited indications. It is also included in the WHO Model List of Essential Medicines that contains the medications considered to be the most effective, safe, and meet the most important needs in a health system [10].

Unfortunately, enterococci evolved a resistance to vancomycin that was first detected in the late 1980s in the United Kingdom and Europe [11, 12].The vancomycin resistance (Van) operon confers resistance to glycopeptides in The Enterococcus species and this operon can be carried on mobile genetic elements and/or chromosomally [13]. One of the components of the Van operon is a variable ligase and, so far, 9 variant genes have been identified [14]. They are classified into two categories based on the ligases they encode: the operons *vanA*,* vanB*,* vanD*, and* vanM*, which encode for D-Ala-D-Lac ligase; and the operons *vanC*,* vanE*,* vanG*,* vanL*, and* vanN* which encodes for the D-Ala-D-Ser ligase [15].

Infections caused by VRE have a great impact on the healthcare system including longer hospital stays, higher death rates, and higher healthcare costs when compared to vancomycin-susceptible enterococci [16, 17]. Furthermore, vancomycin-resistant *E.* *faecium* bacteremia is associated with a bad prognosis and a higher mortality rate than vancomycin-resistant *E.* *faecalis* bacteremia [18, 19].

In the USA, 54,500 estimated cases are hospitalized; 5400 are estimated fatalities. Healthcare costs were estimated to be $539 million in 2017. In 2019, the CDC classified VRE as a serious threat in the United States’ Antibiotic Resistance Threats [20].

Although there are several reports from various Egyptian regions, the pooled incidence of VRE among enterococci clinical isolates in Egypt is unclear. Given the significant impact of VRE on patient mortality and healthcare costs, we conducted a systematic review with meta-analysis to determine the pooled prevalence of VRE among total enterococci clinical isolates, *Enterococcus faecium* and *Enterococcus faecalis* among total enterococci, Vancomycin-resistant *Enterococcus faecalis*, Vancomycin-resistant *Enterococcus faecium*, VanA and VanB genes among VRE, and linezolid, gentamicin, and ampicillin among total enterococci isolates and VRE.

## Methods

### Search strategy

Using the following keywords: enterococci, enterococcus, vancomycin-resistant enterococci, vancomycin-resistant enterococcus, VRE, and Egypt, a thorough literature search was carried out in the following databases: Medline (through PubMed), Scopus, Google Scholar, and Web of Science. Using ZOTERO version 6, search findings were combined, and duplicates were eliminated. Only original publications published in English were included in the search, which was limited to articles published between 2010 and 2022. We followed the Preferred Reporting Items for Systematic Reviews and Meta-Analyses (PRISMA) statement during the preparation of this meta-analysis [21]. The PRISMA Checklist is presented in Fig. S1.

### Inclusion and exclusion criteria

Two independent reviewers (A.Az and M.Y) selected the included studies based on the inclusion and exclusion criteria.

Any study that fulfilled all of the following criteria was included: studies conducted in Egypt; clinical isolates were only included (isolates from patients); studies that reported the prevalence of VRE; and studies using standard methods for detecting VRE. Studies were excluded if they had any of the following: studies published in languages other than English, full text not available, case report studies, review articles, and conference abstracts. Furthermore, articles with fewer than 30 subjects were excluded to reduce any potential bias brought on by a small sample size.

### Data extraction

From each included study, the following data were extracted by two separate reviewers (A.A and H.E) and reviewed by a third (A.A.E): the first author's last name, publication date, government or city, total isolates of enterococci, total count of VRE, *E. faecalis*, and *E. faecium* among total enterococci, Vancomycin-resistant *E. faecalis*, Vancomycin-resistant *E. faecium*, VanA, and VanB genes among VRE, method of detection, specimen (urine/blood/wound, other sources), and resistance to linezolid, high content gentamicin, and ampicillin among total isolates of enterococci and VRE isolates.

### Quality assessment

Two independent reviewers (A.Az and H.K) evaluated the quality of the included studies using a checklist derived from Ding et al. (2017) [22]. Disagreements were settled by consensus.

### Meta-analysis

Statistical analysis has been performed using MedCalc Version 20.113. *I*^2^ and *Q* test were used to measure the heterogeneity between the studies and based on the random effects model, results were reported as proportions with a 95% confidence interval (CI). By visually examining the funnel plot, the risk of bias within studies was assessed. It was then tested using the non-parametric rank test, Begg’s test, and the parametric regression test (also known as “Egger’s test”). Low *p* values (*P* < 0.05) are considered a sign of publication bias in both situations since they show asymmetry. Publication bias testing was not performed when the number of studies was less than 10 [23]. Analyses of the subgroups were conducted based on region and the method used. Sensitivity analysis was conducted by Open Meta-Analyst Software using leave-one-out approach.

## Results

### Characteristics of the included studies

**Fig. 1:**
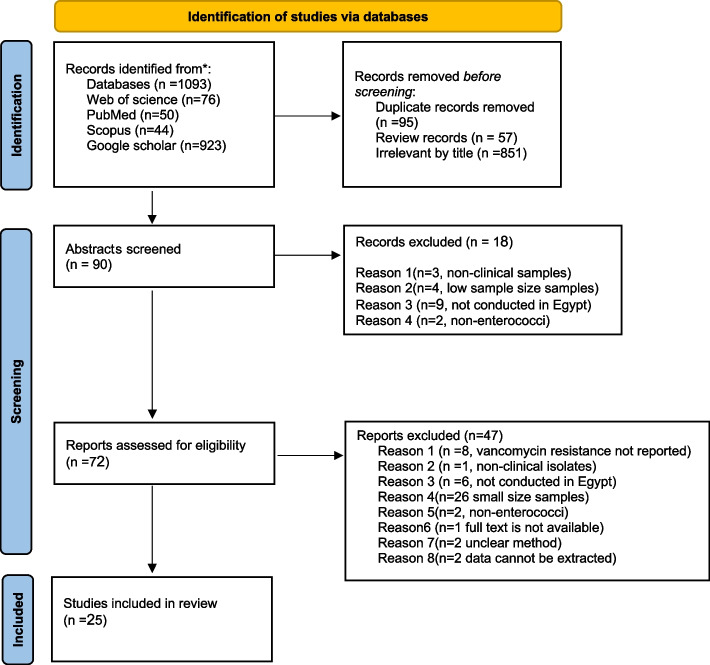
Flow chart depicting the selection of publications

As depicted (Fig. 1), 4 separate databases were searched, yielding a total of 1093 results. Eight hundred fifty-one articles with irrelevant titles, 57 reviews, and 95 duplicates were removed. The remaining 90 publications were then evaluated by reading the abstracts, and 18 were eliminated. By reading the full text, 72 articles were reviewed for eligibility, and by the end, a total of 25 studies fulfilled our inclusion and exclusion criteria and were included in our review [24–48] (Table S1 and S2 summarize the characteristics and the quality of the included studies respectively; see supplementary material).

### The prevalence of VRE among total enterococci clinical isolates

**Fig. 2:**
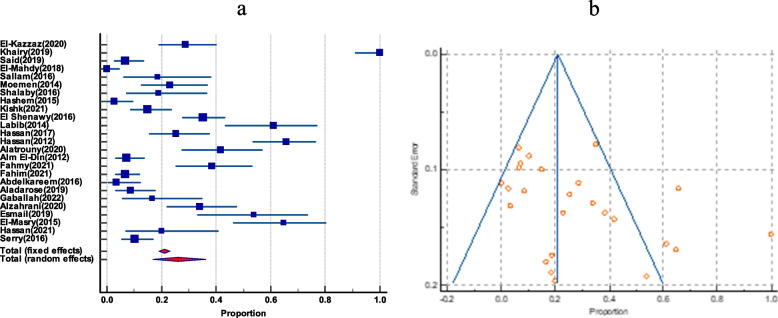
The prevalence of VRE among clinical isolates in Egypt. **a** Forest plot of VRE among total enterococci. **b** Funnel plot of VRE among total enterococci

The pooled prevalence of VRE among enterococci clinical isolates was estimated to be 26% (95% CI 16.9 to 36.3). The studies had a significant degree of heterogeneity, as evident by (*I*^2^ = 95.45%) and Cochrane *Q* test = 528. The funnel plot showed a slight asymmetry by visual inspection; as evidenced by the Egger’s test and Begg’s test these were statistically insignificant (*P* = .0468, and *P* = .0258), respectively (Fig. 2).

### Subgroup analysis

The VRE prevalence was also determined depending on location and antibiotic susceptibility technique. Of 25 studies, 13 studies used the disc diffusion method and 12 studies used the MIC-based methods to determine the susceptibility of enterococci to vancomycin. The results of the meta-analysis based on subgroup were summarized in Table 1.

**Table 1:** Pooled prevalence of VRE in Egypt by subgroups

Subgroup	Included studies	Pooled prevalence(%) and 95% CI	Total number of enterococci	*I*^2^ Heterogeneity	Heterogeneity test, *P* value	Publication bias testing	
						Egger’s test	Begg’s test
**Based on AST**							
** VRE among total enterococci by disk diffusion method**	13	24.02 (11.36 to 39.6)	866	96.05%	*P* < 0.0001	*P* = 0.0480*	*P* = 0.0231*
** VRE among total enterococci by MIC-based methods**	12	28.25 (15.83 to 42.64)	849	94.85%	*P* < 0.0001	*P* = 0.3244	*P* = 0.6286
** VRE among total enterococci by Broth microdilution**	5	26.76 (5.9 to 55.6)	273	95.81%	*P* < 0.0001	N/P^A^	N/P
** VRE among total enterococci by** ***E*** **test**	5	38.24 (24.7 to 52.78)	395	87.60%	*P* < 0.0001	N/P	N/P
** VRE among total enterococci by VITEK 2 Automated System**	3	13 (5.17 to 23.64)	213	66.53%	*P* = 0.0504	N/P	N/P
**Government**							
** Mansoura**	6	12.45 (3.97 to 24.74)	410	90.61%	*P* < 0.0001	N/P	N/P
** Cairo**	6	19.54 (8 to 34.5)	425	91.66%	*P* < 0.0001	N/P	N/P
** Minia**	3	55.5 (0.186 to 99.5)	123	98.74%	*P* < 0.0001	N/P	N/P
** Tanta**	2	12 (3.2 to 25.5)	143	69.93%	*P* = 0.0682	N/P	N/P
** Menoufia**	2	62.5 (51 to 73.3)	70	0.00%	*P* = 0.7605	N/P	N/P
** Zagazig**	2	21.7 (3.4 to 49.8)	289	96.26%	*P* < 0.0001	N/P	N/P
** Sohag**	2	52.4 (26.7 to 77.5)	125	89.08%	*P* = 0.0025	N/P	N/P

^*^Statistically insignificant

^A^Not performed

The pooled VRE prevalence among total enterococci by the disc diffusion method was 24.02% (95% CI 11.36 to 39.6; *I*^2^ = 96.05%; *P* < 0.0001).Visual observation of the funnel plot revealed asymmetry, and there was a statistically significant funnel plot asymmetry as evidenced by Egger’s test and Begg’s test (*P* = 0.0288, *P* = 0.0082) (Figs. S2 and S3; see supplementary material).

On the other hand, the prevalence of VRE based on MIC-based methods was 28.25% (95% CI 15.83 to 42.64; *I*^2^ = 94.85% *P* < 0.0001) and the funnel plot showed symmetry that was evidenced by both Egger’s test and Begg’s test (*P* = 0.9875, *P* = 0.7297, for broth microdilution 26.76% (95% CI 5.9 to 55.6; *I*^2^ = 95.81%; *P* < 0.0001), *E* test 38.24% (95% CI 24.7 to 52.78; *I*^2^ = 87.60%; *P* 0.0001) and for vitek 2 automated system 13% (95% CI 5.17 to 23.64; *I*^2^ = 66.53%; *P* = 0.0504) (Figs. S4–S8; see supplementary material).

In our review, the majority of research was reported from Mansoura (6 studies), Cairo (6 studies), Minia (3), Tanta (2), Menoufia (2), Zagazig (2), Sohag (2), Ismailia (1), and Alexandria and EL Beheira (1).

Table 1 summarizes the prevalence, which ranged from 12.5% (95% CI 3.9 to 24.8; *I*^2^ = 90.61%; *P* 0.0001) and *I*^2^ (95% CI 3.2 to 25.5; *I*^2^ = 69.93%; *P* = 0.0682) for Mansoura and Tanta, respectively, to 62.5% (95% CI 51 to 73.2; *I*^2^ = 0.00%; *P* = 0.7605) for Menoufia (Figs. S9–S15, see supplementary material).

### Prevalence of *E. faecalis* and *E. faecium* among total enterococci

**Fig. 3:**
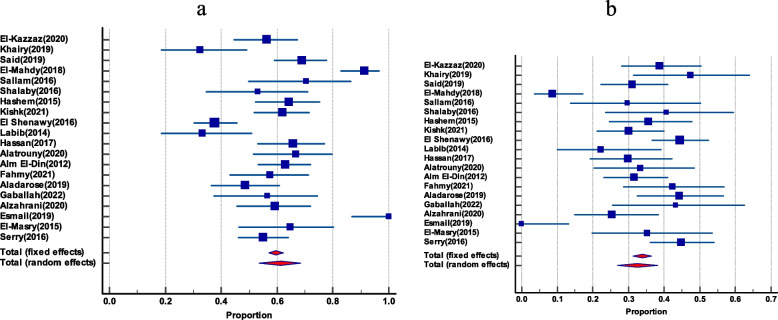
The prevalence of *E. faecalis* and *E. faecium* among total enterococci isolates. **a** Forest plot of *E. faecalis* among total enterococci. **b** Forest plot *E. faecium* among total enterococci

**Fig. 4:**
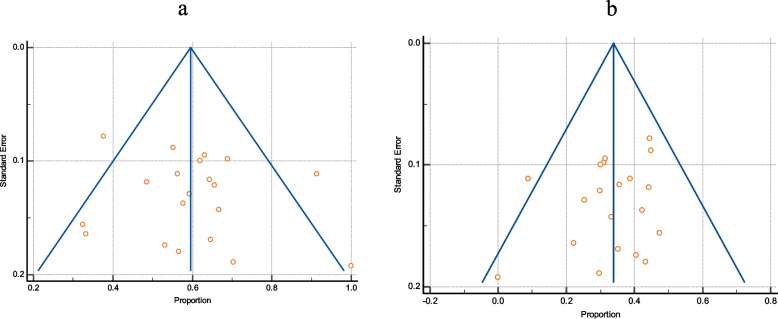
Funnel plot of publication bias for *E. faecalis* and *E. faecium* among total enterococci isolates. **a** Funnel plot of *E. faecalis* among total enterococci. **b** Funnel plot of *E. faecium* among total enterococci

The frequency of *E. faecalis* and *E. faecium* has been co-reported in 20 studies. *E. faecalis* had a greater pooled prevalence than *E. faecium*, with 61.22% (95% CI 53.65 to 68.53) and 32.47% (95% CI 27 to 38.2), respectively. There was no evidence of funnel plot asymmetry by visual inspection of the funnel plot and by both Egger’s test and Begg’s test as shown in (Table 2) and (Figs. 3 and 4).

**Table 2:** Pooled prevalence of *E.* *faecium* and *E.* *faecium* among enterococci, their Vancomycin resistance and VanA and VanB genes among VRE

Group	Included studies	Total number of enterococci	Pooled prevalence(%) and 95% CI	*I*^2^ Heterogeneity	Heterogeneity test, *P* value	Publication bias testing	
						Egger’s test	Begg’s test
***E. faecium* among total enterococci**	20	1357	32.47 (27 to 38.2)	79.47%	*P* < 0.0001	*P* = 0.2349	*P* = 0.3132
***E. faecalis* among total enterococci**	20	1357	61.22 (53.65 to 68.53)	87.58%	*P* < 0.0001	*P* = 0.3485	*P* = 0.5803
**Vancomycin-resistant *E. faecium* among total *E. faecium***	12	266	46.1 (25.7 to 67.1)	79.86%	*P* < 0.0001	*P* = 0.0612	*P* = 0.1120
**Vancomycin-resistant *E. faecalis* among total *E. faecalis***	13	453	31.7 (18.6 to 46.4)	90.53%	*P* < 0.0001	*P* = 0.0091	*P* = 0.0316
**VanA among VRE**	7	192	63.3 (52.1 to 73.7)	57.61%	*P* = 0.0280	N/P^a^	N/P
**VanB genes among VRE**	7	192	17.95 (7.8 to 31)	77.29%	*P* = 0.0002	N/P	N/P

^a^Not performed

### The prevalence of vancomycin-resistant *E. faecalis* among total *E. faecalis*

**Fig. 5:**
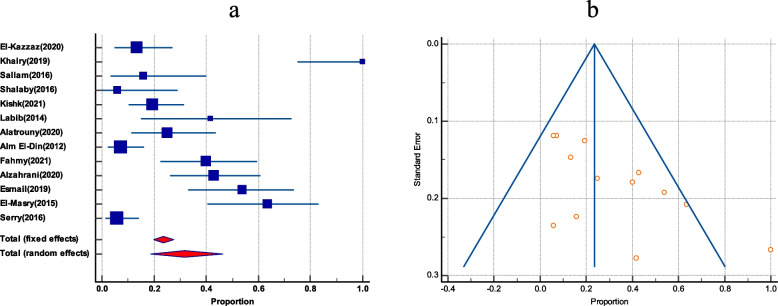
The prevalence of Vancomycin-resistant *E. faecalis among total E. faecalis*. **a** Forest plot Vancomycin-resistant *E. faecalis*. **b** Funnel plot of Vancomycin-resistant *E. faecalis*

In 13 publications, vancomycin-resistant *E. faecalis* was shown to be common among all *E. faecalis*. The pooled prevalence was 31.7% (95% CI 18.6 to 46.4). By visual inspection, the funnel plot displayed asymmetry, which was further confirmed by both Egger’s test and Begg’s test, which showed statistically significant funnel plot asymmetry (as shown in Table 2 and Fig. 5).

### Prevalence of Vancomycin-resistant *E. faecium *among total *E. faecium*

**Fig. 6:**
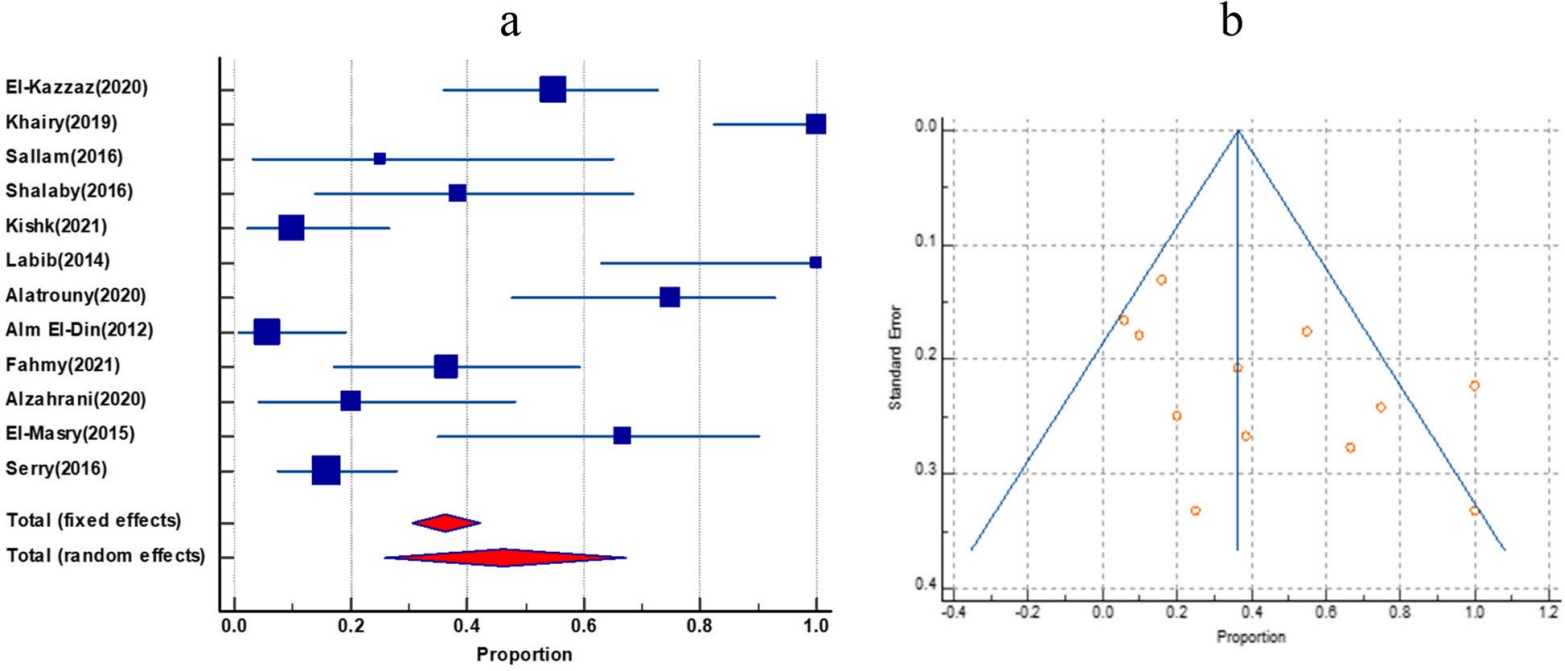
The prevalence of Vancomycin-resistant *E. faecium among total E. faecium.* **a** Forest plot of Vancomycin-resistant *E. faecium.* **b** Funnel plot of Vancomycin-resistant *E. faecium*

Twelve studies gave an account of the prevalence of vancomycin-resistant *E. faecium* among total *E. faecium*. It had a pooled prevalence of 46.1% (95% CI 25.7 to 67.1). Again, there was no evidence of funnel plot asymmetry by visual inspection of the funnel plot and by both Egger’s test and Begg’s test (as presented in Table 2 and Fig. 6).

### VanA and VanB gene among VRE

**Fig. 7:**
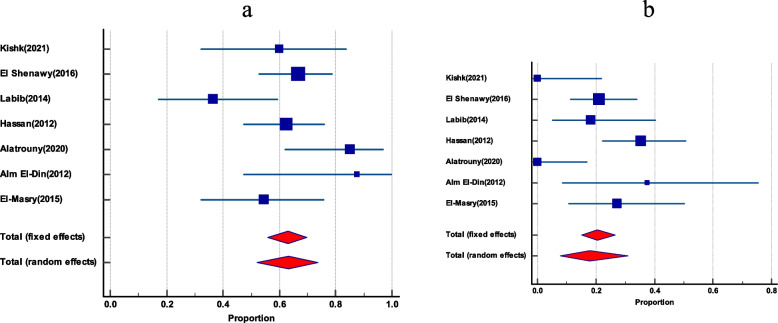
The dissemination of VanA and VanB among VRE. **a** Forest plot of VanA among VRE. **b** Forest plot of VanB gene among VRE

VanA was more frequent than VanB among VRE, with pooled prevalence of 63.3% (95% CI 52.1 to 73.7) and 17.95 (95% CI 7.8 to 31), respectively, as reported by 7 studies that indicate the prevalence of both VanA and VanB genes among VRE (as shown in Fig. 7).

### The resistance profile of enterococci to linezolid, ampicillin, and high content gentamicin

**Fig. 8:**
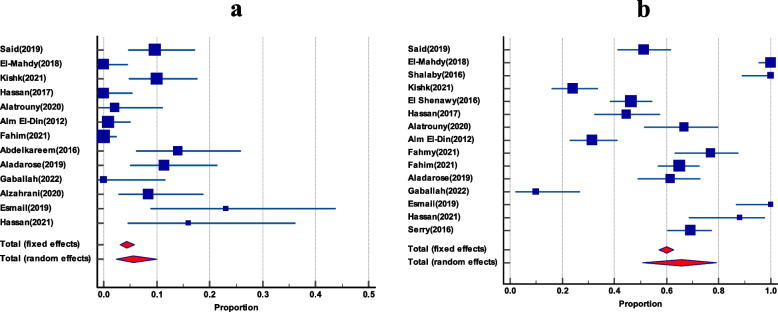
The resistance profile of enterococci isolates to linezolid and ampicillin. **a** Forest plot of linezolid resistance among total enterococci isolates. **b** Forest plot of ampicillin resistance among total enterococci isolates

**Fig. 9:**
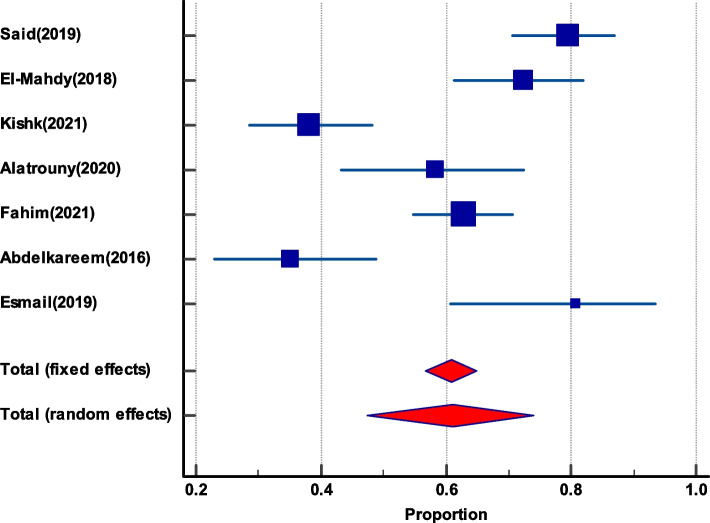
Forest plot of enterococci isolates resistant to high-level gentamicin

**Fig. 10:**
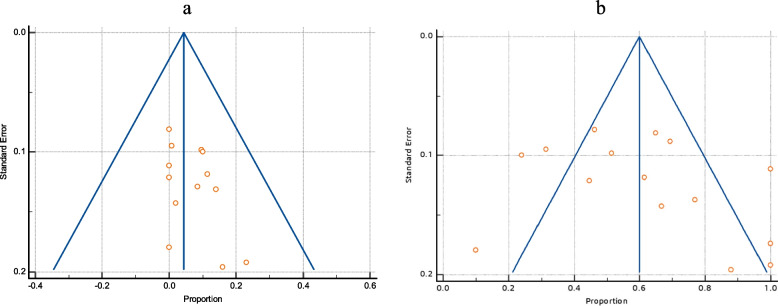
Funnel plots of the resistance profile of the enterococcal isolates to linezolid and ampicillin. **a** Funnel plot of linezolid resistance. **b** Funnel plot of resistance ampicillin

As depicted in Table 3, the pooled resistance rate of linezolid was substantially lower than that of ampicillin and high-level gentamicin (HLG), 5.54% (95% CI 2.33 to 10%), 65.7% (95% CI 50.8 to 79.2%), and 61.1% (95% CI; 47.4 to 73.9%), respectively (Figs. 8 and 9). There was no funnel plot asymmetry (Fig. 10) that was evident by Egger’s test and Begg’s test. (The characteristics of the antibiotic resistance profile among total Enterococci and VRE are summarized in Table S3 and S4; see Supplementary material.)

**Table 3:** Pooled resistance profile of enterococci isolates in Egypt

Group	Included studies	Total number of enterococci	Pooled prevalence(%) and 95% CI	*I*^2^ Heterogeneity	Heterogeneity test, *P* value	Publication bias testing	
						Egger’s test	Begg’s test
**Linezolid resistance among VRE**	4	89	5.2 (1.3 to 11.5%)	15.81%	*P* = 0.3126	N/P	N/P
**Linezolid resistance among total enterococci isolates**	13	927	5.54 (2.33 to 10)	84.82%	*P* < 0.0001	*P* = 0.1249	*P* = 0.1431
**Ampicillin resistance among total enterococci isolates**	15	1184	65.7 (50.8 to 79.2%)	96.39%	*P* < 0.0001	*P* = 0.2124	*P* = 0.1815
**Ampicillin resistance among VRE**	4	140	85 (49 to 100%)	95.33%	*P* < 0.0001	N/P	N/P
**HLG resistance among total enterococci isolates**	7	565	61.1 47.4 to 73.9%)	90.60%	*P* < 0.0001	N/P	N/P

### The resistance profile of VRE to linezolid, and ampicillin

There were only 4 studies that reported linezolid and ampicillin resistance rates among VRE. The pooled resistance rate of linezolid was much lower than that of ampicillin, 5.2% (95% CI 1.3 to 11.5%) and 85% (95% CI 49 to 100%) respectively (Figs. S16 and S17).

### Sensitivity analysis

Sensitivity analysis, using the leave-one-out approach, indicated that the combined estimate of VRE among total enterococci clinical isolates is reliable and is not dependent on any single study (Fig. S18).

## Discussion

To our knowledge, this is the first systematic review and meta-analysis study to evaluate the pooled prevalence of VRE and the antimicrobial resistance profile of enterococci in Egypt. This study is based on data analysis from published literature on the prevalence of VRE in patients in Egypt published between 2010 and 2022. The pooled prevalence of VRE among enterococci clinical isolates in Egypt was estimated to be 26%. *E.* *faecalis* had a greater pooled prevalence than *E.* *faecium*. The VanA gene was more frequent than the VanB gene among VRE. The pooled resistance rate of linezolid was substantially lower than that of ampicillin and high-level gentamicin (HLG).

In our review, the pooled prevalence of VRE among enterococci clinical isolates was 26% (95% CI 16.9 to 36.3), which was higher than the pooled prevalence of VRE among clinical specimens in Iran and Asia, which was 9.4% (95% CI 7.3–12) and 8.10% (95% CI 7–9), respectively [49, 50].

Subgroup analysis based on the antimicrobial susceptibility methods yielded heterogeneous results. The pooled VRE prevalence among total enterococci by the disc diffusion method was 24.02% (11.36 to 39.6) and by MIC-based methods it was 28.25% (95% CI 15.83 to 42.64). The results obtained by vitek 2 were lower than those of the E-test and broth microdilution (9.22% (95% CI 2.69 to 19.1), 26.76% (95% CI 5.9 to 55.6), and 38.24% (95% CI 24.697 to 52.78), but the 95% confidence interval overlapped. These heterogeneous results could be explained by different resistance patterns based on region, specimen source, and technique variability [51].

We also found that the frequency of *E. faecalis* and *E. faecium* has been reported in 20 studies. *E. faecalis* had a greater pooled prevalence than *E. faecium*, with 61.22% (95% CI 53.65 to 68.53) and 32.47% (95% CI 27 to 38.2), respectively. These results were consistent with a meta-analysis by Moghimbeig et al. [52]. However, several reports from the UK, Denmark, the Netherlands, Poland, and Iran have shown a trend towards the replacement of *E. faecalis* by *E. faecium* [53–57].

The prevalence of vancomycin resistance among *E. faecium* and *E. faecalis* was co-reported in 12 studies and showed a higher rate of vancomycin resistance among *E. faecium* than that of *E. faecalis* 46.1% (95% CI 25.7 to 67.1), and 31.7% (95% CI 18.6 to 46.4), respectively, but the 95% CI overlapped. Other studies have also reported similar findings [49, 58, 59]. This point is particularly important as vancomycin-resistant *E. faecium* bacteremia is associated with a bad prognosis and a higher mortality rate than vancomycin-resistant *E. faecalis* bacteremia [18, 19].

According to 7 studies that co-reported the dissemination of VanA and VanB variants, VRE harboring VanA variants were more prevalent than those harboring VanB variants, with a pooled prevalence rate of 63.3% (95%CI 52.1 to 73.7) and 17.95% (95% CI 7.8 to 31), respectively. The dissemination of VanA and VanB variants among enterococci varies worldwide. For instance, VanA is predominant in North America and Europe. On the other hand, the VanB variant is dominant in Australia and New Zealand and is increasingly being reported in Europe [60].

Linezolid is the first member of the oxazolidinone family of antibiotics and is considered one of the last-resort antibiotics for management of VRE infections [61]. the ZAAPS and LEADER surveillance programs, which were set up to monitor linezolid resistance in non-USA and USA countries, respectively, revealed that enterococci were susceptible to linezolid in more than 99% of cases [62, 63]. According to current meta-analysis outcomes, resistance rates of 927 enterococci clinical isolates to linezolid were documented in 13 studies, with a pooled resistance rate of 5.54% (95% CI 2.33 to 10%).The pooled resistance rate of linezolid remained almost consistent against enterococci showing a VRE phenotype similar to the ZAAPS surveillance program [62]. Given this high resistance rate, linezolid should be reserved for treatment of confirmed or suspected infections due to multi-drug-resistant organisms and be de-escalated wherever possible.

Assessing the occurrence of ampicillin and gentamicin resistance in enterococci is clinically important, as a combination of both is recommended in the treatment of ampicillin-sensitive VRE when bactericidal activity is needed [64, 65].

Our study demonstrated a high level of ampicillin resistance among enterococci clinical isolates with a pooled resistance rate of 65.7% (95% CI 50.8 to 79.2%), similar to a study conducted in India that revealed a 75.5% ampicillin resistance rate [66], and much higher than a surveillance study in Europe conducted between 2011 and 2019 that revealed a 10.6% ampicillin resistance rate [67].

High-level gentamicin resistance (HLGR) was documented in seven studies with a pooled resistance rate of 61.1% (95% CI 47.4 to 73.9%). This is somewhat comparable to a meta-analysis done in Iran that revealed a 49.4% (95% CI 42.2 to 56.6%) HLGR to enterococci [68].

Several factors may explain this high prevalence of vancomycin, ampicillin and high-level gentamicin resistance among enterococci isolates in Egypt. First, infection control programs are not very adequate in Egypt. Workload, inadequate resources, limited opportunities for infection control training and insufficient staff were the most common obstacles complained about by healthcare workers against the practice of standard precautions [69–73]. Second, the inappropriate use of antibiotics and antibiotic self-medication are prevalent in Egypt [74–76].

We think the following measures may be needed to limit further increases in antibiotic resistance among enterococci or other pathogens. First, a national Antimicrobial Resistance Policy in Egypt to understand the emergence, spread, and factors influencing antimicrobial resistance. Second, a prohibition on antibiotic self-medication. Third, efforts to educate healthcare workers and patients about the proper use of antimicrobials. Fourth, rapid molecular diagnostics to support appropriate antimicrobial use. Fifth, if not previously established, infection control strategies and antimicrobial stewardship practices should be followed. Sixth, research is needed to define “inappropriate” antimicrobial prescribing and to better understand the primary drivers of such use.

There are some limitations to our study. First, our results do not fully reflect the prevalence of VRE in Egypt, as not all regions in Egypt reported the prevalence of VRE. Second, there was a high heterogeneity in VRE prevalence between studies that could stem from the difference in antibiotic resistance pattern from region to region or from AST methods themselves. Third, the small number of included studies is of concern.

## Conclusion

Given the high incidence of resistance to vancomycin, linezolid, high-level gentamicin, and ampicillin in clinical specimens from enterococci in Egypt, we strongly advise that healthcare settings develop and follow their own antibiogram to guide choosing an appropriate empirical therapy as well as implementing infection control programs to prevent further escalation of the problem.

## Supplementary Information

**Additional file 1: Figure S1.** Supplementary Preferred Reporting Items for Systematic Reviews and Meta-analyses (PRISMA) checklist. **Table S1.** Characteristics of the included studies. **Table S2.** the quality of included studies. **Table S3.** Characteristics of the antibiotic resistance profile among total Enterococci isolates. **Table S4.** Characteristics of the antibiotic resistance profile of linezolid and ampicillin among vancomycin-resistant enterococci (VRE). **Figure S2.** Forest plot of VRE among total enterococci by disc diffusion method. **Figure S3.** Funnel plot of VRE among total enterococci by disc diffusion method. **Figure S4.** Forest plot of VRE among total enterococci by MIC-based methods. **Figure S5.** Funnel plot of VRE among total enterococci by MIC-based methods. **Figure S6.** Forest plot of VRE among total enterococci by broth Microdilution. **Figure S7.** Forest plot of VRE among total enterococci by viteck 2 automated system. **Figure S8.** Forest plot of VRE among total enterococci by E-test. **Figure S9.** Forest plot of VRE in Mansoura. **Figure S10.** Forest plot of VRE in Cairo. **Figure S11.** Forest plot of VRE in Minia. **Figure S12.** Forest plot of VRE in Sohag. **Figure S13.** Forest plot of VRE in Tanta. **Figure S14.** Forest plot of VRE in Menofia. **Figure S15.** Forest plot of VRE in Zagazig. **Figure S16.** Forest plot of linezolid resistance among VRE. **Figure S17.** Forest plot ampicillin resistance among VRE. **Figure S18.** Forest plot of leave-one-out meta-analysis with random effect for the prevalence of VRE among clinical isolates in Egypt.

## Data Availability

All data generated or analyzed during this study are included in this published article [and its supplementary information file].We have no conflicts of interest to disclose.

## Abbreviations

VRE: Vancomycin-resistant Enterococci
CDC: Centers for Disease Control and Prevention
HLG: High level gentamicin
PRISMA: Preferred Reporting Items for Systematic Reviews and Meta-Analyses

## References

[1] Van Boeckel TP, Brower C, Gilbert M, Grenfell BT, Levin SA, Robinson TP (2015). Global trends in antimicrobial use in food animals. Proc Natl Acad Sci U S A.

[2] Ma YX, Wang CY, Li YY, Li J, Wan QQ, Chen JH (2020). Considerations and caveats in combating ESKAPE pathogens against nosocomial infections. Adv Sci.

[3] Khan HA, Ahmad A, Mehboob R (2015). Nosocomial infections and their control strategies. Asian Pac J Trop Biomed.

[4] Fiore E, Van Tyne D, Gilmore MS. Pathogenicity of enterococci. Microbiol Spectr. 2019;7(4). 10.1128/microbiolspec.gpp3-0053-2018 10.1128/microbiolspec.gpp3-0053-2018 PMC6629438 31298205

[5] Parte AC, Carbasse JS, Meier-Kolthoff JP, Reimer LC, Göker M (2020). List of prokaryotic names with standing in nomenclature (LPSN) moves to the DSMZ. Int J Syst Evol Microbiol.

[6] Cattoir V (2022). The multifaceted lifestyle of enterococci: genetic diversity, ecology and risks for public health. Curr Opin Microbiol.

[7] Franz CMAP, Huch M, Abriouel H, Holzapfel W, Gálvez A (2011). Enterococci as probiotics and their implications in food safety. Int J Food Microbiol.

[8] Franz CMAP, Stiles ME, Schleifer KH, Holzapfel WH (2003). Enterococci in foods—a conundrum for food safety. Int J Food Microbiol.

[9] Ghahremani M, Jazani NH, Sharifi Y (2018). Emergence of vancomycin-intermediate and -resistant Staphylococcus aureus among methicillin-resistant S. aureus isolated from clinical specimens in the northwest of Iran. J Glob Antimicrob Resist.

[10] World Health Organization, Medicines Selection, IP and Affordability. WHO 2021 AWaRe classification. 2021. Available from: https://www.who.int/publications/i/item/2021-aware-classification; https://www.who.int/westernpacific/publications-detail/2021-aware-classification%0A. Accessed 21 Oct 2022.

[11] Lam MMC, Seemann T, Bulach DM, Gladman SL, Chen H, Haring V (2012). Comparative analysis of the first complete Enterococcus faecium genome. J Bacteriol.

[12] Werner G, Coque TM, Hammerum AM, Hope R, Hryniewicz W, Johnson A (2008). Emergence and spread of vancomycin resistance among enterococci in Europe. Euro Surveill.

[13] Ahmed MO, Baptiste KE (2018). Vancomycin-resistant enterococci: a review of antimicrobial resistance mechanisms and perspectives of human and animal health. Microb Drug Resist.

[14] Walsh CT, Fisher SL, Park IS, Prahalad M, Wu Z (1996). Bacterial resistance to vancomycin: five genes and one missing hydrogen bond tell the story. Chem Biol.

[15] George SK, Suseela MR, El Safi S, Elnagi EA, Al-Naam YA, Adam AM (2021). Molecular determination of van genes among clinical isolates of enterococci at a hospital setting. Saudi J Biol Sci.

[16] DiazGranados CA, Zimmer SM, Klein M, Jernigan JA (2005). Comparison of mortality associated with vancomycin-resistant and vancomycin-susceptible enterococcal bloodstream infections: a meta-analysis. Clin Infect Dis.

[17] Cheah ALY, Spelman T, Liew D, Peel T, Howden BP, Spelman D (2013). Enterococcal bacteraemia: factors influencing mortality, length of stay and costs of hospitalization. Clin Microbiol Infect.

[18] Hayakawa K, Marchaim D, Martin ET, Tiwari N, Yousuf A, Sunkara B (2012). Comparison of the clinical characteristics and outcomes associated with vancomycin-resistant Enterococcus faecalis and vancomycin-resistant *E.*
*faecium* bacteremia. Antimicrob Agents Chemother.

[19] Ghanem G, Hachem R, Jiang Y, Chemaly RF, Raad I (2007). Outcomes for and risk factors associated with vancomycin-resistant Enterococcus faecalis and vancomycin-resistant Enterococcus faecium bacteremia in cancer patients. Infect Control Hosp Epidemiol.

[20] Centers for Disease Control and Prevention (CDC). 2019 antibiotic resistance threats report. 2021. Available from: https://www.cdc.gov/drugresistance//biggest-threats.html. Accessed 21 Oct 2022.

[21] Moher D, Liberati A, Tetzlaff J, Altman DG (2009). Preferred reporting items for systematic reviews and meta-analyses: the PRISMA statement. BMJ.

[22] Ding H, Gao YM, Deng Y, Lamberton PHL, Lu DB (2017). A systematic review and meta-analysis of the seroprevalence of Toxoplasma gondii in cats in mainland China. Parasit Vectors.

[23] Higgins JPT, Green S. Cochrane Handbook for Systematic Reviews of Interventions: 10.4.3.1 Recommendations on testing for funnel plot asymmetry. 2011. Available from: https://handbook-5-1.cochrane.org/chapter_10/10_4_3_1_recommendations_on_testing_for_funnel_plot_asymmetry.htm. Accessed 21 Oct 2022.

[24] Serry F, Elmasry E, Heagazy W, Abdel-Karim S (2016). Antibiotic resistance of Enterococcus faecalis and Enterococcus faecium isolated from urinary tract infections in Zagazig University hospitals. Zagazig J Pharm Sci.

[25] Hassan R, El-Gilany AH, Abd-Elaal AM, El-Mashad N, Abdelazim D. Antibiotic resistance pattern of bacteria causing hospital acquired infections in the New Mansoura general hospital, Egypt. Arch Community Med 2021;3(1). 10.36959/547/645

[26] Alaa-Eldin RA, El-mahdy HS (2012). Molecular characterization of enterococcus strains isolated from cases of neonatal sepsis in neonatal intensive care unit. African J Microbiol Res.

[27] Alatrouny AMM, Amin MA, Shabana HS (2020). Prevalence of vancomycin resistant enterococci among patients with nosocomial infections in intensive care unit. Al-Azhar Med J.

[28] Hassan A, Fattouh M, El-deen A, Maguid‏ S. Isolation and characterization of vancomycin-resistant enterococci (Vre) in surgical wards of Sohag University Hospital. Egy J Med Lab Sci. 2012;21(2):101–11.

[29] Hassan RM, Ghaith DM, Ismail DK, Zafer MM. Reduced susceptibility of Enterococcus spp. isolates from Cairo University Hospital to tigecycline: highlight on the influence of proton pump inhibitors. J Glob Antimicrob Resist. 2018;12:68–72. 10.1016/j.jgar.2017.12.005. 10.1016/j.jgar.2017.12.005 29274469

[30] Azza L, Ahmed M, Nahed AR, Wafaa Z, Eman E (2013). Molecular and phenotypic characterization of hospital-associated and community-associated isolates of Enterococcus spp. Menoufia Med J.

[31] El Shenawy GA, Abdel-Latif RS, Shedeed DS (2016). Detection of VanA, VanB and VanC genes in vancomycin resistant enterococci in Zagazig University Hospitals. Egypt J Med Microbiol.

[32] Kishk R, Nemr N, Soliman N, Riad E, Ahmed M, Soliman N (2021). High-level Aminoglycoside and Vancomycin resistance in Enterococcus spp. Isolated from Hospital Acquired Infections,&nbsp;Ismailia Egypt. Egypt J Med Microbiol.

[33] Hashem YA, Yassin AS, Amin MA (2015). Molecular characterization of Enterococcus spp. clinical isolates from Cairo, Egypt. Indian J Med Microbiol.

[34] Shalaby MM, Kareman AE, Wageih SEN, El-Sharaby RM (2016). Comparative study between molecular and non-molecular methods used for detection of Vancomycin Resistant Enterococci in Tanta University Hospitals. Egypt Life Sci J.

[35] Moemen D, Tawfeek D, Badawy W (2015). Healthcare-associated vancomycin resistant enterococcus faecium infections in the Mansoura university hospitals intensive care units. Egypt Brazilian J Microbiol.

[36] El-Masry EA, Awad ET, Yassin MH (2015). Antibiotic resistance, molecular typing, biofilm formation in Enterococcai isolates causing urinary tract infection. J Pure Appl Microbiol.

[37] Sallam MM, Abou-Aisha K, El-Azizi M (2016). A novel combination approach of human polyclonal IVIG and antibiotics against multidrug-resistant gram-positive bacteria. Infect Drug Resist.

[38] El-Mahdy R, Mostafa A, El-Kannishy G (2018). High level aminoglycoside resistant enterococci in hospital-acquired urinary tract infections in Mansoura. Egypt Germs.

[39] Said HS, Abdelmegeed ES (2019). Emergence of multidrug resistance and extensive drug resistance among enterococcal clinical isolates in Egypt. Infect Drug Resist.

[40] Khairy RM, Mahmoud MS, Esmail MAM, Gamil AN (2019). First detection of vanB phenotype-vanA genotype vancomycin-resistant enterococci in Egypt. J Infect Dev Ctries.

[41] El-Kazzaz SS, Abou El-Khier NT (2020). Effect of the lantibiotic nisin on inhibitory and bactericidal activities of antibiotics used against vancomycin-resistant enterococci. J Glob Antimicrob Resist.

[42] Esmail MAM, Abdulghany HM, Khairy RM (2019). Prevalence of multidrug-resistant *Enterococcus **faecalis* in hospital-acquired surgical wound infections and bacteremia: concomitant analysis of antimicrobial resistance Genes. Infect Dis Res Treat.

[43] Alzahran NH, Mohamed EA (2020). Evaluation of the antibacterial and anticancer activities of marine Bacillus subtilis ESRAA3010 against different multidrug resistant Enterococci (MDRE) and cancer cell lines. Arch Biotechnol Biomed.

[44] Gaballah A, Shawky S, Amer A (2022). Microbiological profiles of neonatal sepsis in northern Egypt. Microbes Infect Dis.

[45] Aladarose BE, Said HS, Abdelmegeed ES (2019). Incidence of virulence determinants among enterococcal clinical isolates in Egypt and its association with biofilm formation. Microb Drug Resist.

[46] Abdelkareem MZ, Sayed M, Hassuna NA, Mahmoud MS, Abdelwahab SF (2017). Multi-drug-resistant Enterococcus faecalis among Egyptian patients with urinary tract infection. J Chemother.

[47] Fahim NAE (2021). Prevalence and antimicrobial susceptibility profile of multidrug-resistant bacteria among intensive care units patients at Ain Shams University Hospitals in Egypt—a retrospective study. J Egypt Public Health Assoc.

[48] Fahmy N, Abdel-Gawad A, Rezk G, Mahmoud E. Characterization of Enterococci isolated from intensive care unit (ICU): distribution of virulence markers, virulence genes and antibiotic resistance pattern. Microbes Infect Dis. 2021;2(4):725–35. 10.21608/mid.2021.76391.1158.

[49] Emaneini M, Hosseinkhani F, Jabalameli F, Nasiri MJ, Dadashi M, Pouriran R, Beigverdi R (2016). Prevalence of vancomycin-resistant Enterococcus in Iran: a systematic review and meta-analysis. Eur J Clin Microbiol Infect Dis.

[50] Shrestha S, Kharel S, Homagain S, Aryal R, Mishra SK (2021). Prevalence of vancomycin-resistant enterococci in Asia—A systematic review and meta-analysis. J Clin Pharm Ther.

[51] Kohner PC, Patel R, Uhl JR, Garin KM, Hopkins MK, Wegener LT (1997). Comparison of agar dilution, broth microdilution, E-test, disk diffusion, and automated vitek methods for testing susceptibilities of Enterococcus spp. to vancomycin. J Clin Microbiol.

[52] Moghimbeigi A, Moghimbeygi M, Dousti M, Kiani F, Sayehmiri F, Sadeghifard N (2018). Prevalence of vancomycin resistance among isolates of enterococci in Iran: a systematic review and meta-analysis. Adolesc Health Med Ther.

[53] Sattari-Maraji A, Jabalameli F, Node Farahani N, Beigverdi R, Emaneini M (2019). Antimicrobial resistance pattern, virulence determinants and molecular analysis of Enterococcus faecium isolated from children infections in Iran. BMC Microbiol.

[54] Gawryszewska I, Żabicka D, Bojarska K, Malinowska K, Hryniewicz W, Sadowy E (2016). Invasive enterococcal infections in Poland: the current epidemiological situation. Eur J Clin Microbiol Infect Dis.

[55] Top J, Willems R, Blok H, De Regt M, Jalink K, Troelstra A (2007). Ecological replacement of Enterococcus faecalis by multiresistant clonal complex 17 Enterococcus faecium. Clin Microbiol Infect.

[56] Lester CH, Sandvang D, Olsen SS, Schønheyder HC, Jarløv JO, Bangsborg J (2008). Emergence of ampicillin-resistant Enterococcus faecium in Danish hospitals. J Antimicrob Chemother.

[57] Horner C, Mushtaq S, Allen M, Hope R, Gerver S, Longshaw C (2021). Replacement of Enterococcus faecalis by Enterococcus faecium as the predominant enterococcus in UK bacteraemias. JAC-Antimicrobial Resist.

[58] Zhou W, Zhou H, Sun Y, Gao S, Zhang Y, Cao X (2020). Characterization of clinical enterococci isolates, focusing on the vancomycin-resistant enterococci in a tertiary hospital in China: based on the data from 2013 to 2018. BMC Infect Dis.

[59] Orababa OQ, Soriwei JD, Akinsuyi SO, Essiet UU, Solesi OM (2021). A systematic review and meta-analysis on the prevalence of vancomycin-resistant enterococci (VRE) among Nigerians. Porto Biomed J.

[60] Lee T, Pang S, Abraham S, Coombs GW (2019). Molecular characterization and evolution of the first outbreak of vancomycin-resistant Enterococcus faecium in Western Australia. Int J Antimicrob Agents.

[61] Bender JK, Cattoir V, Hegstad K, Sadowy E, Coque TM, Westh H (2018). Update on prevalence and mechanisms of resistance to linezolid, tigecycline and daptomycin in enterococci in Europe: towards a common nomenclature. Drug Resist Updat.

[62] Mendes RE, Deshpande L, Streit JM, Sader HS, Castanheira M, Hogan PA (2018). ZAAPS programme results for 2016: an activity and spectrum analysis of linezolid using clinical isolates from medical centres in 42 countries. J Antimicrob Chemother.

[63] Flamm RK, Mendes RE, Hogan PA, Streit JM, Ross JE, Jonesa RN (2016). Linezolid surveillance results for the United States (LEADER Surveillance Program 2014). Antimicrob Agents Chemother.

[64] Arias CA, Contreras GA, Murray BE (2010). Management of multidrug-resistant enterococcalinfections. Clin Microbiol Infect.

[65] O’Driscoll T, Crank CW (2015). Vancomycin-resistant enterococcal infections: epidemiology, clinical manifestations, and optimal management. Infect Drug Resist.

[66] Yadav G, Thakuria B, Madan M, Agwan V, Pandey A (2017). Linezolid and vancomycin resistant enterococci: a therapeutic problem. J Clin Diagn Res.

[67] Hrbacek J, Cermak P, Zachoval R (2020). Current antibiotic resistance trends of uropathogens in central Europe: survey from a Tertiary Hospital Urology Department 2011–2019. Antibiot.

[68] Abadi MSS, Taji A, Salehi F, Kazemian H, Heidari H (2021). High-level gentamicin resistance among clinical isolates of enterococci in Iran: a systematic review and meta-analysis. Folia Med (Plovdiv).

[69] Refeai SA, Kamal NN, Ghazawy ERA, Fekry CM (2020). Perception and barriers regarding infection control measures among healthcare workers in Minia City. Egypt. Int J Prev Med.

[70] Greeb HEE, Ahmed AI, Atia HM, Mouty SMA. Assessment of nurses’ compliance with infection control standard precautions at outpatient clinics of urology and nephrology center - Mansoura University. J Nurs Heal Sci. 2018;7(3):54–9. http://www.iosrjournals.org/iosr-jnhs/papers/vol7-issue3/Version-1/G0703015459.pdf. Accessed 21 Oct 2022.

[71] Salem MR, Youssef MRL (2017). Health care providers’ perspectives for providing quality infection control measures at the neonatal intensive care unit, Cairo University Hospital. Am J Infect Control.

[72] Salam MESA, El-Shazly HMA, Dewidar MAAS (2014). Infection control awareness among healthcare providers in family health settings in Shebin El-kom district, Menoufia Governorate. Egypt Menoufia Med J.

[73] Hendy A, Al-Sharkawi S, Hassanein SMA, Soliman SM. Effect of educational intervention on nurses’ perception and practice of antimicrobial stewardship programs. Am J Infect Control. 10.1016/J.AJIC.2022.05.001. 10.1016/j.ajic.2022.05.001 35561943

[74] Elsayed AA, Darwish SF, Zewail MB, Mohammed M, Saeed H, Rabea H (2021). Antibiotic misuse and compliance with infection control measures during COVID-19 pandemic in community pharmacies in Egypt. Int J Clin Pract.

[75] Aly MM, Elchaghaby MA (2021). The prescription pattern and awareness about antibiotic prophylaxis and resistance among a group of Egyptian pediatric and general dentists: a cross sectional study. BMC Oral Health.

[76] El-Hawy RM, Ashmawy MI, Kamal MM, Khamis HA, Abo El-Hamed NM, Eladely GI (2017). Studying the knowledge, attitude and practice of antibiotic misuse among Alexandria population. Eur J Hosp Pharm.

