# Supplementary material for: Prevalence of Vancomycin-resistant enterococci (VRE) in Egypt (2010–2022): a systematic review and meta-analysis
Source: J Egypt Public Health Assoc. 2023 Apr 11;98:8. doi: 10.1186/s42506-023-00133-9 (PMC10086090; doi:10.1186/s42506-023-00133-9)
Supplement: Supplementary file 1 — Additional file 1: Figure S1. Supplementary Preferred Reporting Items for Systematic Reviews and Meta-analyses (PRISMA) checklist. Table S1. Characteristics of the included studies. Table S2. the quality of included studies. Table S3. Characteristics of the antibiotic resistance profile among total Enterococci isolates. Table S4. Characteristics of the antibiotic resistance profile of linezolid and ampicillin among vancomycin-resistant enterococci (VRE). Figure S2. Forest plot of VRE among total enterococci by disc diffusion method. Figure S3. Funnel plot of VRE among total enterococci by disc diffusion method. Figure S4. Forest plot of VRE among total enterococci by MIC-based methods. Figure S5. Funnel plot of VRE among total enterococci by MIC-based methods. Figure S6. Forest plot of VRE among total enterococci by broth Microdilution. Figure S7. Forest plot of VRE among total enterococci by viteck 2 automated system. Figure S8. Forest plot of VRE among total enterococci by E-test. Figure S9. Forest plot of VRE in Mansoura. Figure S10. Forest plot of VRE in Cairo. Figure S11. Forest plot of VRE in Minia. Figure S12. Forest plot of VRE in Sohag. Figure S13. Forest plot of VRE in Tanta. Figure S14. Forest plot of VRE in Menofia. Figure S15. Forest plot of VRE in Zagazig. Figure S16. Forest plot of linezolid resistance among VRE. Figure S17. Forest plot ampicillin resistance among VRE. Figure S18. Forest plot of leave-one-out meta-analysis with random effect for the prevalence of VRE among clinical isolates in Egypt. [file 42506_2023_133_MOESM1_ESM.docx]

**Prevalence of Vancomycin-resistant enterococci (VRE) in Egypt: a systematic review and meta-analysis**

Ahmed Azzam ^1^, Hoda Elkafas ^2^, Heba Khaled ^3^, Ahmed Ashraf ^4^, Mohammed Abd-Ellatif Yousef ^5^, Aya Awny Elkashef ^6^

^1^ Department of Microbiology and Immunology, Faculty of Pharmacy, Helwan University, Cairo Egypt.
^2^ Department of Pharmacology and Toxicology, Egyptian Drug Authority, formerly National Organization for Drug Control and Research, Cairo 35521, Egypt.
^3^ Department of biochemistry and molecular biology, Faculty of Pharmacy, Cairo University, Egypt.
^4^ Faculty of pharmacy Minia University, Minia, Egypt.
^5^ Faculty of pharmacy Al-Azhar University, Assuit, Egypt.
^6^ Department of Botany & Microbiology, Faculty of Science, Arish University, North Sinai, Egypt.

**Fig.S1: Supplementary Preferred Reporting Items for Systematic Reviews and Meta-analyses (PRISMA) checklist**

| **Section and Topic** | **Item #** | **Checklist item** | **Location where item is reported** |
| --- | --- | --- | --- |
| **TITLE** | | |  |
| Title | 1 | Identify the report as a systematic review. | P.1 |
| **ABSTRACT** | | |  |
| Abstract | 2 | See the PRISMA 2020 for Abstracts checklist. | P.2 |
| **INTRODUCTION** | | |  |
| Rationale | 3 | Describe the rationale for the review in the context of existing knowledge. | P.4 |
| Objectives | 4 | Provide an explicit statement of the objective(s) or question(s) the review addresses. | P.4 |
| **METHODS** | | |  |
| Eligibility criteria | 5 | Specify the inclusion and exclusion criteria for the review and how studies were grouped for the syntheses. | P.5-6 |
| Information sources | 6 | Specify all databases, registers, websites, organisations, reference lists and other sources searched or consulted to identify studies. Specify the date when each source was last searched or consulted. | P.5 |
| Search strategy | 7 | Present the full search strategies for all databases, registers and websites, including any filters and limits used. | P.5 |
| Selection process | 8 | Specify the methods used to decide whether a study met the inclusion criteria of the review, including how many reviewers screened each record and each report retrieved, whether they worked independently, and if applicable, details of automation tools used in the process. | P.5 |
| Data collection process | 9 | Specify the methods used to collect data from reports, including how many reviewers collected data from each report, whether they worked independently, any processes for obtaining or confirming data from study investigators, and if applicable, details of automation tools used in the process. | P.5-6 |
| Data items | 10a | List and define all outcomes for which data were sought. Specify whether all results that were compatible with each outcome domain in each study were sought (e.g. for all measures, time points, analyses), and if not, the methods used to decide which results to collect. | P.5-6 |
|  | 10b | List and define all other variables for which data were sought (e.g. participant and intervention characteristics, funding sources). Describe any assumptions made about any missing or unclear information. | P.5-6 |
| Study risk of bias assessment | 11 | Specify the methods used to assess risk of bias in the included studies, including details of the tool(s) used, how many reviewers assessed each study and whether they worked independently, and if applicable, details of automation tools used in the process. | P.6 |
| Effect measures | 12 | Specify for each outcome the effect measure(s) (e.g. risk ratio, mean difference) used in the synthesis or presentation of results. | P.6 |
| Synthesis methods | 13a | Describe the processes used to decide which studies were eligible for each synthesis (e.g. tabulating the study intervention characteristics and comparing against the planned groups for each synthesis (item #5)). | Table S1 |
|  | 13b | Describe any methods required to prepare the data for presentation or synthesis, such as handling of missing summary statistics, or data conversions. | - |
|  | 13c | Describe any methods used to tabulate or visually display results of individual studies and syntheses. | P.6 |
|  | 13d | Describe any methods used to synthesize results and provide a rationale for the choice(s). If meta-analysis was performed, describe the model(s), method(s) to identify the presence and extent of statistical heterogeneity, and software package(s) used. | P.6 |
|  | 13e | Describe any methods used to explore possible causes of heterogeneity among study results (e.g. subgroup analysis, meta-regression). | P.6 |
|  | 13f | Describe any sensitivity analyses conducted to assess robustness of the synthesized results. |  |
| Reporting bias assessment | 14 | Describe any methods used to assess risk of bias due to missing results in a synthesis (arising from reporting biases). | P.6 |
| Certainty assessment | 15 | Describe any methods used to assess certainty (or confidence) in the body of evidence for an outcome. | P.6 |
| **RESULTS** | | |  |
| Study selection | 16a | Describe the results of the search and selection process, from the number of records identified in the search to the number of studies included in the review, ideally using a flow diagram. | Fig.1 |
|  | 16b | Cite studies that might appear to meet the inclusion criteria, but which were excluded, and explain why they were excluded. | P.7 |
| Study characteristics | 17 | Cite each included study and present its characteristics. | Table S1 |
| Risk of bias in studies | 18 | Present assessments of risk of bias for each included study. | Table 1-3 |
| Results of individual studies | 19 | For all outcomes, present, for each study: (a) summary statistics for each group (where appropriate) and (b) an effect estimate and its precision (e.g. confidence/credible interval), ideally using structured tables or plots. | Fig.(2-10) |
| Results of syntheses | 20a | For each synthesis, briefly summarise the characteristics and risk of bias among contributing studies. | Table 1-3 |
|  | 20b | Present results of all statistical syntheses conducted. If meta-analysis was done, present for each the summary estimate and its precision (e.g. confidence/credible interval) and measures of statistical heterogeneity. If comparing groups, describe the direction of the effect. | Table 1-3 |
|  | 20c | Present results of all investigations of possible causes of heterogeneity among study results. | Table 1 |
|  | 20d | Present results of all sensitivity analyses conducted to assess the robustness of the synthesized results. | P.10 and Fig. S18 |
| Reporting biases | 21 | Present assessments of risk of bias due to missing results (arising from reporting biases) for each synthesis assessed. | Table S2 |
| Certainty of evidence | 22 | Present assessments of certainty (or confidence) in the body of evidence for each outcome assessed. | - |
| **DISCUSSION** | | |  |
| Discussion | 23a | Provide a general interpretation of the results in the context of other evidence. | P.14-16 |
|  | 23b | Discuss any limitations of the evidence included in the review. | P.17 |
|  | 23c | Discuss any limitations of the review processes used. | P.17 |
|  | 23d | Discuss implications of the results for practice, policy, and future research. | P.16 |
| **OTHER INFORMATION** | | |  |
| Registration and protocol | 24a | Provide registration information for the review, including register name and registration number, or state that the review was not registered. | Not prepared |
|  | 24b | Indicate where the review protocol can be accessed, or state that a protocol was not prepared. | Not prepared |
|  | 24c | Describe and explain any amendments to information provided at registration or in the protocol. | - |
| Support | 25 | Describe sources of financial or non-financial support for the review, and the role of the funders or sponsors in the review. | P.17 |
| Competing interests | 26 | Declare any competing interests of review authors. | P.17 |
| Availability of data, code and other materials | 27 | Report which of the following are publicly available and where they can be found: template data collection forms; data extracted from included studies; data used for all analyses; analytic code; any other materials used in the review. | P.17 |

Abbreviations: p: page.

| Table S1 Characteristics of the included studies | | | | | | | | | | | | | | | | |
| --- | --- | --- | --- | --- | --- | --- | --- | --- | --- | --- | --- | --- | --- | --- | --- | --- |
|  |  |  |  |  |  |  | Genes among VRE | | species | | | specimen | | | |  |
| First author and publication year | **Government /city** | **Total enterococci** | **Total of VRE** | **method** | **VRE faecalis** | **VRE faecium** | **VanA** | **VanB** | ***E. faecalis*** | ***E. faecium*** | **Other species** | **urine** | **blood** | **wound** | **Other sources** | **references** |
| El-Kazzaz(2020) | Mansoura | 80 | 23 | E-test | 6 | 17 | - | - | 45 | 31 | 4 | 38 | 28 | 14 | 0 | ^1^ |
| Khairy(2019) | Minia | 40 | 40 | disc diffusion method | 13 | 19 | 32 | - | 13 | 19 | 8 | - | 5 | 35 | - | ^2^ |
| Said(2019) | Mansoura | 103 | 7 | broth  microdilution method | - | - | 0 | - | 71 | 32 | - | 56 | 36 | 11 | - | ^3^ |
| El-Mahdy(2018) | Mansoura | 80 | 0 | disc diffusion | - | - | - | - | 73 | 7 |  | 80 | - | - | - | ^4^ |
| Sallam(2016) | Cairo | 27 | 5 | Broth micro dilution | 3 | 2 | - | - | 19 | 8 | - | - | - | - | - | ^5^ |
| Moemen(2014) | Mansoura | 52 | 12 | disc diffusion | - | - | - | - | - | 52 | - | N/S* | N/S * | N/S * | - | ^6^ |
| Shalaby(2016) | Tanta | 32 | 6 | E-test  and vitek 2^£^ | 1 | 5 | 6 | - | 17 | 13 | 2 | 13 | 6 | 9 | 4(endotracheal aspirates | ^7^ |
| Hashem(2015) | Cairo | 73 | 2 | Broth micro dilution | - | - | 2 | - | 47 | 26 | 0 | N/S* | - | - | - | ^8^ |
| Kishk(2021) | Ismailia | 100 | 15 | disk diffusion | 12 | 3 | 9 | 0 | 62 | 30 | 8 | 42 | 6 | 33 | 19 | ^9^ |
| El Shenawy(2016) | Zagazig | 162 | 57 | E-test |  |  | 38 | 12 | 61 | 72 | 29 | 95 | 0 | 26 | 41 | ^10^ |
| Labib(2014) | Menofia | 36 | 22 | broth dilution method | 5 | 8 | 8 | 4 | 12 | 8 | 16 | N/S* | N/S* | - | Pus and sputum | ^11^ |
| Hassan(2017) | Cairo | 67 | 17 | disk diffusion | - | - | - | - | 44 | 20 | 3 | 44 | 6 | 12 | 1 | ^12^ |
| Hassan(2012) | sohag | 73 | 48 | E-test | 11 | 33 | 30 | 17 |  |  | - | N/S* | - | - | pus | ^13^ |
| Alatrouny(2020) | Cairo | 48 | 20 | E-test method | 8 | 12 | 17 | 0 | 32 | 16 | - | 8 | 20 | 12 | - | ^14^ |
| Alm El-Din(2012) | Tanta | 111 | 8 | disc-diffusion method | 5 | 2 | 7 | 3 | 70 | 35 | 6 | - | 111 | - | - | ^15^ |
| Fahmy(2021) | Sohag | 52 | 20 | disk diffusion method | 12 | 8 |  |  | 30 | 22 | - | N/S* | N/S* | - | Pus  and sputum | ^16^ |
| Fahim(2021) | Cairo | 151 | 10 | Vitek 2/ agar dilution method | - | - | - | - | - | - | - | 74 | 64 | 13 | - | ^17^ |
| Abdelkareem(2016) | Minia | 57 | 2 | disk  diffusion method | - | - | - | - | - | - | - | 57 | - | - | - | ^18^ |
| Aladarose(2019) | Mansoura | 70 | 6 | disc  diffusion method | - | - | - | - | 34 | 31 | 5 | 33 | 12 | 10 | 15 | ^19^ |
| Gaballah(2022) | Alexandria and  EL Behira | 30 | 5 | vitek 2 | - | - | - | - | 17 | 13 | - | - | 30 | - | - | ^20^ |
| Alzahrani(2020) | Cairo | 59 | 20 | disk diffusion | 15 | 3 | - | - | 35 | 15 | 9 | 12 | 9 | 10 | 28 | ^21^ |
| Esmail(2019) | Minia | 26 | 14 | disk  diffusion method | 14 | 0 | 10 | - | 26 | 0 | 0 | - | 2 | 24 | - | ^22^ |
| El-Masry(2015) | Menofia | 34 | 22 | broth dilution | 14 | 8 | 12 | 6 | 22 | 12 | 0 | 34 | - | - | - | ^23^ |
| Hassan(2021) | Mansoura | 25 | 5 | disk diffusion | - | - | - | - | - | - | - | N/S* | N/S* | - | surgical  site  swabs, bronchial aspirate | ^24^ |
| Serry(2016) | Zagazig | 127 | 13 | disc diffusion | 4 | 9 | - | - | 70 | 57 | 0 | 127 | - | - | - | ^25^ |

***=Not specified**

**£ = both e-test and vitek results gave the same results**

**Table S2 the quality of included studies***

| author and publication year | Was the research objective clearly described and stated? | Was the sampling method described in detail? | Was the period and location of the study clearly stated? | Were the examination method and procedure for VRE detection clearly pointed out? | Were the samples clearly classified into different subgroups? | Total score (%) |
| --- | --- | --- | --- | --- | --- | --- |
| El-Kazzaz(2020) | 2 | 1 | 2 | 2 | 2 | **90** |
| Khairy(2019) | 2 | 1 | 2 | 2 | 2 | **90** |
| Said(2019) | 2 | 1 | 2 | 2 | 0 | **70** |
| El-Mahdy(2018) | 2 | 1 | 2 | 2 | 0 | **70** |
| Sallam(2016) | 2 | 1 | 0 | 2 | 2 | **70** |
| Moemen(2014) | 2 | 2 | 2 | 2 | 0 | **80** |
| Shalaby(2016) | 2 | 2 | 0 | 2 | 2 | **80** |
| Hashem(2015) | 2 | 1 | 0 | 2 | 0 | **50** |
| Kishk(2021) | 2 | 1 | 0 | 2 | 2 | **70** |
| El Shenawy(2016) | 2 | 2 | 2 | 2 | 0 | **80** |
| Labib(2014) | 2 | 2 | 0 | 2 | 2 | **80** |
| Hassan(2017) | 2 | 2 | 2 | 2 | 0 | **80** |
| Hassan(2012) | 2 | 2 | 2 | 2 | 2 | **100** |
| Alatrouny(2020) | 2 | 1 | 2 | 2 | 0 | **70** |
| Alm El-Din(2012) | 2 | 2 | 2 | 2 | 2 | **100** |
| Fahmy(2021) | 2 | 1 | 2 | 2 | 2 | **90** |
| Fahim(2021) | 2 | 1 | 2 | 2 | 0 | **70** |
| Abdelkareem(2016) | 2 | 2 | 2 | 2 | 2 | **100** |
| Aladarose(2019) | 2 | 1 | 2 | 2 | 0 | **70** |
| Gaballah(2022) | 2 | 2 | 2 | 2 | 0 | **80** |
| Alzahrani(2020) | 2 | 2 | 0 | 2 | 0 | **60** |
| Esmail(2019) | 2 | 2 | 2 | 2 | 2 | **100** |
| El-Masry(2015) | 2 | 1 | 2 | 2 | 2 | **90** |
| Hassan(2021) | 2 | 1 | 2 | 2 | 0 | **70** |
| Serry(2016) | 2 | 2 | 0 | 2 | 2 | **80** |

* The quality of the included studies were evaluated using a checklist derived from Ding et al. (2017).
The checklist had the following five questions: (1) was the research objective clearly described and stated? (2) Was the sampling method described in detail? (3) Was the period and location of the study clearly stated? (4) Were the examination method and procedure for VRE detection clearly pointed out? (5) Were the samples clearly classified into different subgroups?).
Each question was scored using a basic scale method ("2" for yes, "0" for no, and "1" for unsure).

**Table S3 Characteristics of the antibiotic resistance profile among total Enterococci isolates.**

| first author (publication year) | Total number of enterococci  isolates | Number of resistant isolates of enterococci | | |  |  |
| --- | --- | --- | --- | --- | --- | --- |
|  |  | **linezolid** | **gentamicin (high content)** | **ampicillin** | **method** | **references** |
| Said(2019) | **103** | **10** | **82** | **53** | **disc diffusion** | ^3^ |
| El-Mahdy(2018) | **80** | **0** | **58** | **80** | **disc diffusion** | ^4^ |
| Shalaby(2016) | **32** | **-** | **-** | **32** | **disc diffusion** | ^7^ |
| Kishk(2021) | **100** | **10** | **38** | **24** | **disc diffusion** | ^9^ |
| El Shenawy(2016) | **162** | **-** | **-** | **75** | **disc diffusion** | ^10^ |
| Hassan(2017) | **67** | **0** | **-** | **30** | **disc diffusion** | ^12^ |
| Alatrouny(2020) | **48** | **1** | **28** | **32** | **disc diffusion** | ^14^ |
| Alm El-Din(2012) | **111** | **1** | **-** | **35** | **disc diffusion** | ^15^ |
| Fahmy(2021) | **52** | **-** | **-** | **40** | **disc diffusion** | ^16^ |
| Fahim(2021) | **151** | **0** | **95** | **98** | **disc diffusion** | ^17^ |
| Abdelkareem(2016) | **57** | **8** | **20** | **-** | **disc diffusion** | ^18^ |
| Aladarose(2019) | **70** | **8** |  | **43** | **disc diffusion** | ^19^ |
| Gaballah(2022) | **30** | **0** | **-** | **3** | **disc diffusion** | ^20^ |
| Alzahrani(2020) | **59** | **5** | **-** | **-** | **disc diffusion** | ^21^ |
| Esmail(2019) | **26** | **6** | **21** | **26** | **disc diffusion** | ^22^ |
| Hassan(2021) | **25** | **4** | **-** | **22** | **disc diffusion** | ^24^ |
| Serry(2016) | **127** | **-** | **-** | **88** | **disc diffusion** | ^25^ |

**Table S4: Characteristics of the antibiotic resistance profile of linezolid and ampicillin among vancomycin-resistant enterococci (VRE).**

| first author (publication year) | Total number of VRE | resistance isolates of VRE | |  |  |
| --- | --- | --- | --- | --- | --- |
|  |  | **linezolid** | **ampicillin** | **method** | **references** |
| El-Kazzaz(2020) | **23** | **3** | **17** | **disc diffusion** | ^1^ |
| Moemen(2014) | **12** | **0** | **12** | **disc diffusion** | ^6^ |
| Shalaby(2016) | **6** | **0** | **-** | **disc diffusion** | ^7^ |
| El Shenawy(2016) | **57** | **-** | **27** | **disc diffusion** | ^10^ |
| Hassan(2012) | **48** | **1** | **48** | **disc diffusion** | ^13^ |

**Fig.S2**


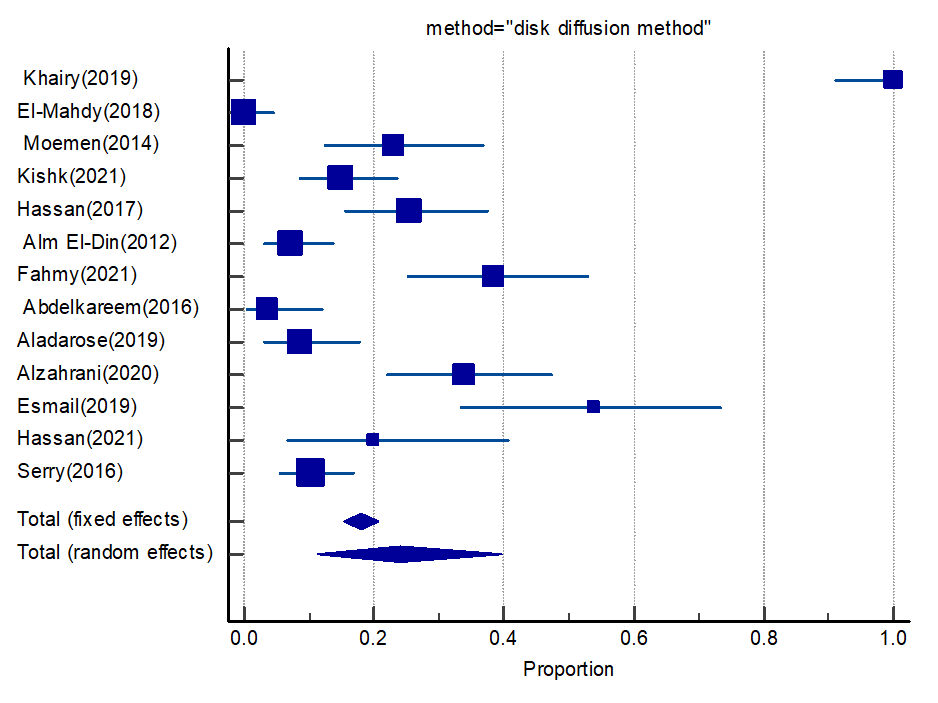


Forest plot of VRE among total enterococci by disc diffusion method

**Fig. S3**


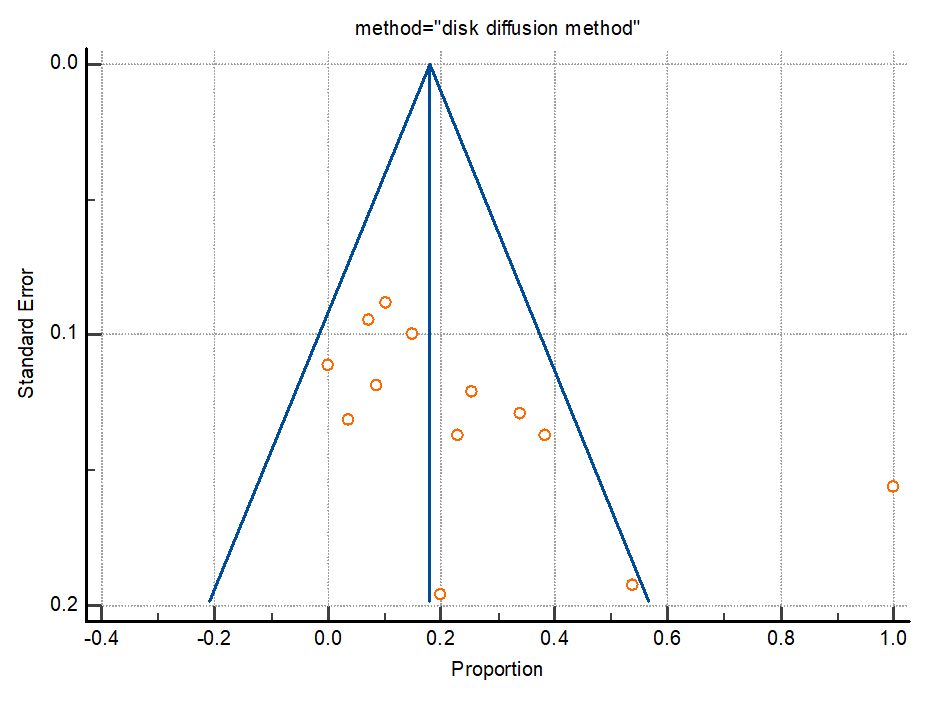


Funnel plot of VRE among total enterococci by disc diffusion method

**Fig. S4**

Forest plot of VRE among total enterococci by MIC-based methods

**Fig. S5**

Funnel plot of VRE among total enterococci by MIC-based methods

**Fig. S6**


forest plot of VRE among total enterococci by broth Microdilution

**Fig. S7**

forest plot of VRE among total enterococci by viteck 2 automated system

**Fig. S8**

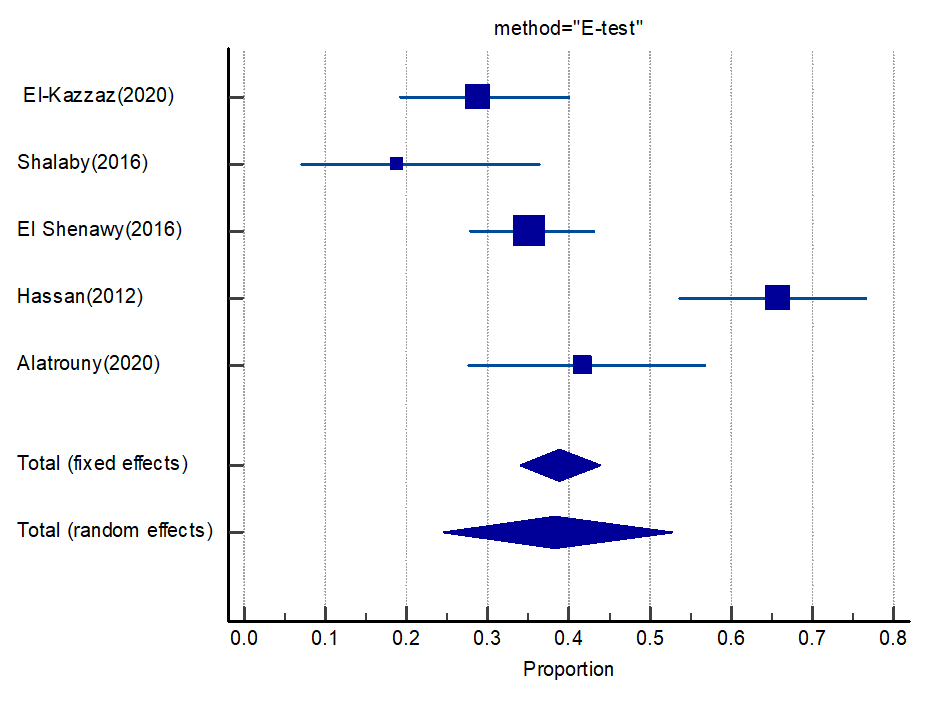


Forest plot of VRE among total enterococci by E-test

**Fig. S9**


 Forest plot of VRE in Mansoura

**Fig. S10**

Forest plot of VRE in Cairo

**Fig. S11**

Forest plot of VRE in Minia

**Fig. S12**

Forest plot of VRE in Sohag

**Fig. S13**

Forest plot of VRE in Tanta

**Fig. S14**

Forest plot of VRE in Menofia

**Fig. S15**

Forest plot of VRE in Zagazig

**Fig. S16**


Forest plot of linezolid resistance among VRE

Fig. S17

Forest plot ampicillin resistance among VRE

**Fig.S18**

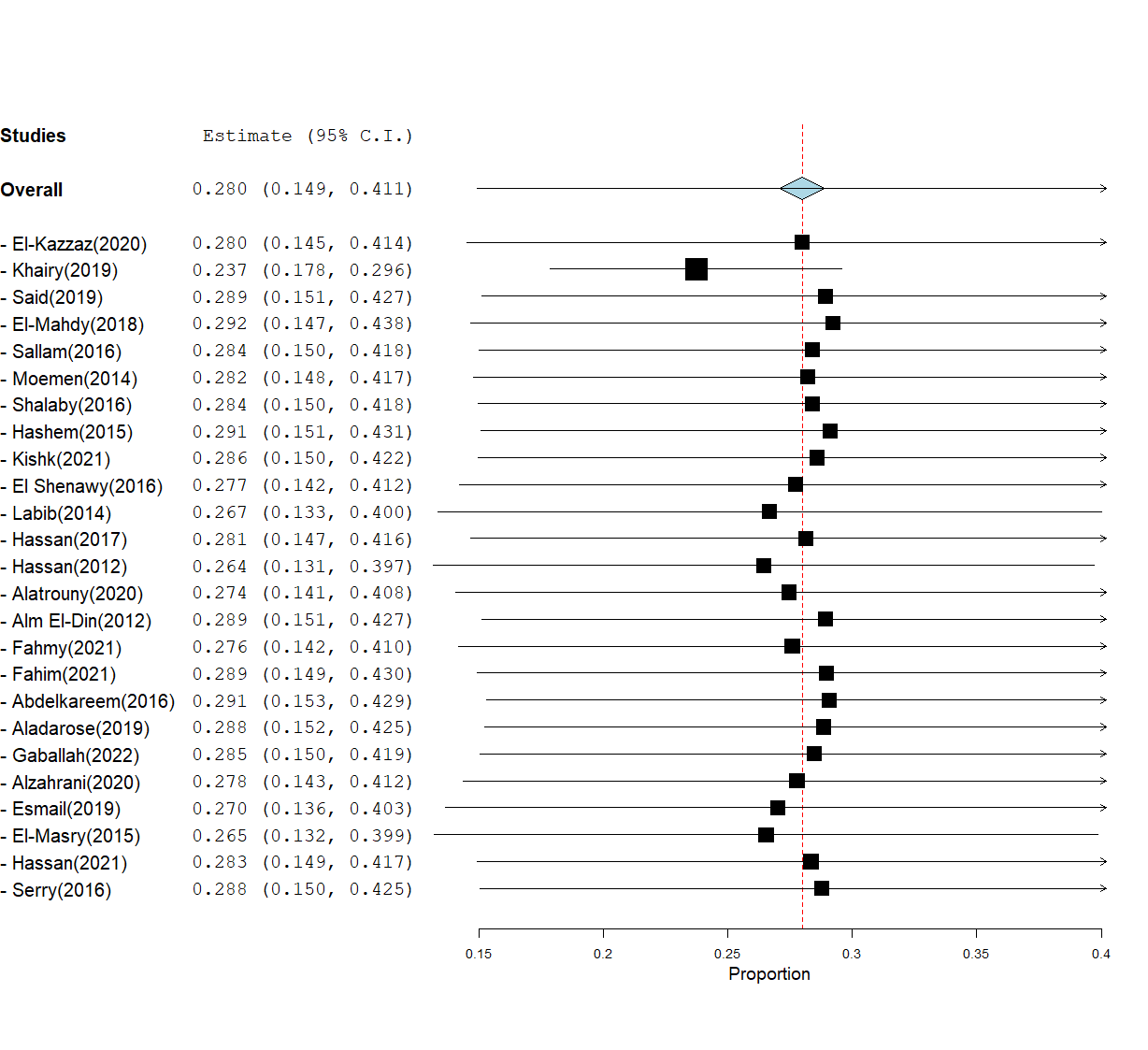


Forest plot of leave-one-out meta-analysis with random effect for the prevalence of VRE among clinical isolates in Egypt

**References**

1. El-Kazzaz SS, Abou El-Khier NT. Effect of the lantibiotic nisin on inhibitory and bactericidal activities of antibiotics used against vancomycin-resistant enterococci. *J Glob Antimicrob Resist*. 2020;22:263-69. doi:10.1016/j.jgar.2020.02.031

2. Khairy RM, Mahmoud MS, Esmail MAM, Gamil AN. First detection of vanB phenotype-vanA genotype vancomycin-resistant enterococci in Egypt. *J Infect Dev Ctries*. 2019;13(9):837-42. doi:10.3855/jidc.10472

3. Said HS, Abdelmegeed ES. Emergence of multidrug resistance and extensive drug resistance among enterococcal clinical isolates in Egypt. *Infect Drug Resist*. 2019;12:1113-25. doi:10.2147/IDR.S189341

4. El-Mahdy R, Mostafa A, El-Kannishy G. High level aminoglycoside resistant enterococci in hospital-acquired urinary tract infections in Mansoura, Egypt. *Germs*. 2018;8(4):186-90. doi:10.18683/germs.2018.1145

5. Sallam MM, Abou-Aisha K, El-Azizi M. A novel combination approach of human polyclonal IVIG and antibiotics against multidrug-resistant gram-positive bacteria. *Infect Drug Resist*. 2016;9:301-11. doi:10.2147/IDR.S120227

6. Moemen D, Tawfeek D, Badawy W. Healthcare-associated vancomycin resistant enterococcus faecium infections in the Mansoura university hospitals intensive care units, Egypt. *Brazilian J Microbiol*. 2015;46(3):777-83. doi:10.1590/S1517-838246320140403

7. Shalaby MM, Kareman AE, Wageih SEN, El-Sharaby RM. Comparative study between molecular and non-molecular methods used for detection of Vancomycin Resistant Enterococci in Tanta University Hospitals, Egypt. Life Sci J 2016;13(1s):71-78. doi:10.7537/marslsj1301s1608

8. Hashem YA, Yassin AS, Amin MA. Molecular characterization of Enterococcus spp. clinical isolates from Cairo, Egypt. *Indian J Med Microbiol*. 2015;33:S80-86. doi:10.4103/0255-0857.148836

9. Kishk R, Nemr N, Soliman N, Riad E, Ahmed M, Soliman N. High-Level Aminoglycoside and Vancomycin Resistance in Enterococcus spp. Isolated from Hospital Acquired Infections, Ismailia, Egypt. *Egypt J Med Microbiol*. 2021;0(0):0-0. doi:10.21608/ejmm.2021.94566.1006

10. El Shenawy GA, Abdel-Latif RS, Shedeed DS. Detection of VanA, VanB and VanC Genes in Vancomycin Resistant Enterococci in Zagazig University Hospitals. *Egypt J Med Microbiol*. 2016;25(1):141-48. doi:10.12816/0037103

11. Azza L, Ahmed M, Nahed AR, Wafaa Z, Eman E. Molecular and phenotypic characterization of hospital-associated and community-associated isolates of Enterococcus spp. *Menoufia Med J*. 2013;26(2):108. doi:10.4103/1110-2098.126138

12. Hassan RM, Ghaith DM, Ismail DK, Zafer MM. Reduced susceptibility of Enterococcus spp. isolates from Cairo University Hospital to tigecycline: Highlight on the influence of proton pump inhibitors. *J Glob Antimicrob Resist*. 2018;12:68-72. doi:10.1016/j.jgar.2017.12.005

13. Hassan A, Fattouh M, El-deen A, Maguid‏ S. Isolation and Characterization of Vancomycin-Resistant Enterococci (Vre) in Surgical Wards of Sohag University Hospital. *Egy j Med Lab Sci*. Published online 2012.

14. Alatrouny AMM, Amin MA, Shabana HS. Prevalence of Vancomycin Resistant Enterococci Among Patients With Nosocomial Infections in Intensive Care Unit. Al-Azhar Med J 2020;49(4):1955-64. doi:10.21608/amj.2020.120651

15. El-din RAA, El-mahdy HS. Molecular characterization of enterococcus strains isolated from cases of neonatal sepsis in neonatal intensive care unit. *African J Microbiol Res*. 2012;6(44):7206-211. doi:10.5897/AJMR12.1144

16. fahmy nahed, Abdel-Gawad A, Rezk G, Mahmoud E. Characterization of Enterococci isolated from intensive care unit (ICU); Distribution of virulence markers, virulence genes and antibiotic resistance pattern. *Microbes Infect Dis*. 2021;0(0):0-0. doi:10.21608/mid.2021.76391.1158

17. Fahim NAE. Prevalence and antimicrobial susceptibility profile of multidrug-resistant bacteria among intensive care units patients at Ain Shams University Hospitals in Egypt—a retrospective study. *J Egypt Public Health Assoc*. 2021;96(1). doi:10.1186/s42506-020-00065-8

18. Abdelkareem MZ, Sayed M, Hassuna NA, Mahmoud MS, Abdelwahab SF. Multi-drug-resistant Enterococcus faecalis among Egyptian patients with urinary tract infection. *J Chemother*. 2017;29(2):74-82. doi:10.1080/1120009X.2016.1182358

19. Aladarose BE, Said HS, Abdelmegeed ES. Incidence of Virulence Determinants among Enterococcal Clinical Isolates in Egypt and Its Association with Biofilm Formation. *Microb Drug Resist*. 2019;25(6):880-89. doi:10.1089/MDR.2018.0320

20. Gaballah A, Shawky S, Amer A. Microbiological profiles of neonatal sepsis in northern Egypt. *Microbes Infect Dis*. 2022;0(0):0-0. doi:10.21608/mid.2022.129600.1265

21. Alzahran NH, Mohamed EA. Evaluation of the antibacterial and anticancer activities of marine Bacillus subtilis ESRAA3010 against different multidrug resistant Enterococci (MDRE) and cancer cell lines. Arch Biotechnol Biomed 2020;4(1):018-27. doi:10.29328/journal.abb.1001018

22. Esmail MAM, Abdulghany HM, Khairy RM. Prevalence of Multidrug-Resistant Enterococcus faecalis in Hospital-Acquired Surgical Wound Infections and Bacteremia: Concomitant Analysis of Antimicrobial Resistance Genes . *Infect Dis Res Treat*. 2019;12:117863371988292. doi:10.1177/1178633719882929

23. El-Masry EA, Awad ET, Yassin MH. Antibiotic resistance, molecular typing, biofilm formation in Enterococcai isolates causing urinary tract infection. *J Pure Appl Microbiol*. 2015;9(3):1859-66.

24. Hassan R, El-Gilany AH, Abd-Elaal AM, El-Mashad N, Abdelazim D. Antibiotic Resistance Pattern of Bacteria Causing Hospital Acquired Infections in the New Mansoura General Hospital, Egypt. Arch Community Med 2021;3(1). doi:10.36959/547/645

25. Serry F, Elmasry E, Heagazy W, Abdel-Karim S. Antibiotic resistance of Enterococcus faecalis and Enterococcus faecium isolated from urinary tract infections in Zagazig University hospitals. *Zagazig J Pharm Sci*. 2016;25(1):30-8. doi:10.21608/zjps.2016.38137
